# Management of Acute Lower Limb Ischemia Without Surgical Revascularization – A Population-Based Study

**DOI:** 10.1177/15385744231215552

**Published:** 2023-11-08

**Authors:** Stefan Acosta, Andrea Kulezic, Moncef Zarrouk, Anders Gottsäter

**Affiliations:** 1 Department of Clinical Sciences, 174435 Lund University, Malmö, Sweden; 2 Vascular Center, Department of Cardiothoracic and Vascular Surgery, Skåne University Hospital, Malmö, Sweden; 3 Department of Acute and Internal Medicine, Skåne University Hospital, Malmö, Sweden

**Keywords:** acute lower limb ischemia, population-based study, conservative therapy, anticoagulation, major amputation

## Abstract

**Objectives:**

To evaluate outcomes of management without surgical revascularization in patients with acute lower limb ischemia (ALI) in a population-based setting.

**Design:**

Retrospective observational population-based study.

**Materials:**

Patients from Malmö, Sweden, hospitalized for ALI between 2015 and 2018.

**Methods:**

In-hospital, surgical, radiological, and autopsy registries were scrutinized for descriptive data on ALI patients managed by endovascular and open vascular surgery, conservative vascular therapy, primary major amputation, and palliative care.

**Results:**

Among 161 patients, 73 (45.3%) did not undergo any operative revascularization. Conservative vascular therapy, primary amputation, and palliative care were conducted in 25 (15.5%), 26 (16.1%), and 22 (13.7%) patients, respectively. Conservatively treated patients had Rutherford class ≥ IIb ischemia and embolic occlusion in 33% and 68% of cases, respectively. Their median C-reactive protein level at admission was 7 mg/L (interquartile range 2 – 31 mg/L). Among conservatively treated patients, anticoagulation therapy in half to full dose was given to 22 (88%) patients for six weeks or longer, and analgesics in low or moderate doses were given to twelve (48%) patients at discharge. The major amputation rate at 1 year was 8% among conservatively treated patients, and four patients with foot embolization had not undergone amputation at 1 year.

**Conclusion:**

Patients selected for initial conservative therapy of ALI with anticoagulation alone may have a good outcome, even when admitted with Rutherford class IIb ischemia. A low C-reactive protein level at admission seems to be a favorable marker when choosing conservative therapy. A prospective, preferably multicenter, study with a predefined protocol in these conservatively treated patients is warranted to better define the dose and length of anticoagulation therapy.

## Introduction

Acute lower limb ischemia (ALI) requires urgent recognition and management as it threatens limb survival and life.^1^ ALI may be treated by open, endovascular, or hybrid revascularization, conservative therapy with anticoagulation alone, amputation, or palliative measures.^2^

Nationwide large-scale epidemiological studies relying on administrative hospital claims data have drawbacks such as miscoding between ALI and chronic limb-threatening ischemia and uncertainty regarding the etiology of ischemia; embolism or in-situ thrombosis.^3^ Vascular registries^4^ do not include the unknown proportion of patients managed by non-operative medical revascularization, primary major amputation, or palliative care, and population-based studies on patients with ALI^5,6^ are rarely conducted. A recent population-based study showed that 45% did not undergo an attempt for operative revascularization,^7^ and this large subgroup of patients with ALI merits a more in-depth evaluation of outcomes.

The aim of this study was therefore to evaluate the outcome of management without surgical revascularization in patients with ALI in a population-based setting.

## Materials and Methods

### Setting

Skåne University Hospital is the only hospital in Malmö, and its Vascular Centre is a tertiary referral center for peripheral vascular disease. Malmö had a population of 338,582 in 2018, 170,987 women and 167,595 men (Statistics Sweden; www.scb.se), and the autopsy rate in the population was 13.6%.^7^

### Inclusion and Exclusion Criteria

This is a retrospective observational study in which patients with ALI registered as living in Malmö, Sweden, between 1st January 2015 and 31st December 2018 were identified. All patients with aorto-iliac or infrainguinal thromboembolic occlusions caused by embolism or thrombosis, occluded lower limb bypass or endoprosthesis, or thromboembolism secondary to popliteal artery aneurysm were included. The following patients were excluded: Patients with ALI in the upper extremities, acute or elective operations for aortic aneurysm or other indications complicated by ALI, elective operations requiring thrombo-embolectomy during or after the primary operation, aortic type A or B dissections with ALI, thrombotic occlusions of the femoral artery with ALI after percutaneous endovascular intervention, iatrogenic vascular injuries resulting in ALI, and traumatic arterial injuries or occlusions with ALI.

### Retrieval of Patients With Acute Lower Limb Ischemia

Hospital charts of all patients with a possible diagnosis of ALI retrieved from the different patient information systems were evaluated.^7^ Data on patient characteristics, comorbidities, medical therapy, laboratory data, severity of ALI, arterial occlusion characteristics, treatment, and complications were collected, and each patient was followed in the electronic patient data system until major amputation, death, or end of follow up August 20th, 2021. There was no missing data for major amputation or mortality. Mortality was checked against the National Population Registry.

### Classification of Native Artery Occlusion

The type of native artery occlusion, embolic or thrombotic, was determined by the appearance of the occlusion and the extent of atherosclerotic wall lesions at computed tomography angiography,^8^ magnetic resonance angiography, or angiography. Embolic occlusions often appear as an oval-shaped clot surrounded by contrast in a non-calcified arterial segment, whereas a thrombotic occlusion usually appears as a clot superimposed on a heavily calcified occlusive lesion.^8^ Presence of synchronous embolism, atrial fibrillation, previous embolism, and no or inadequate anticoagulation therapy at onset of ALI suggested embolism.^9^ In case of an indeterminate cause of the clot and when was considered necessary to optimize medical treatment in conservatively treated patients, Holter electrocardiogram, echocardiography, and extended laboratory testing for vasculitis or arterial thrombophilia were often performed in the outpatient setting.

### Antibiotic Prophylaxis in Major Amputations

According to local guidelines, cloxacillin 2 g intravenously is given three times per day perioperatively, where the first dose is given 30 minutes prior to start of the operation. For infected and necrotic foot wounds, a more broad-spectrum antibiotic prophylaxis is chosen, such as cefotaxime 1 g intravenously three times per day, Guidance of intravenous perioperative antibiotic prophylaxis is based upon available preoperative positive wound cultures and bacterial resistance patterns.

### Definitions

ALI was defined as a symptom duration of less than two weeks.^10^ Ischemic heart disease was defined as patients with previous myocardial infarction, angina, coronary artery bypass grafting (CABG), or percutaneous transluminal coronary angioplasty (PTCA). Hypertension was defined as systolic blood pressure ≥140 mmHg or diastolic blood pressure ≥90 mmHg,^11^ previously known hypertension, or use of antihypertensive medication. The Rutherford classification of ischemia is defined as resting pain (I), sensory loss (IIa), motor deficit (IIb), and irreversible damage with paralysis (III).^12^ Anemia was defined as hemoglobin <134 g/L in men and <117 g/L in women. Renal insufficiency was defined as creatinine >105 μmol/L in men and >90 μmol/L in women. Major amputation was defined as amputation above the ankle. The patient cohort was divided into five treatment groups; endovascular therapy, open vascular surgery, conservative vascular therapy, primary major amputation, and palliative therapy. The presence of postoperative wound infection of the amputation stump was based on clinical judgement and wound cultures. Major bleeding was defined as bleeding necessitating transfusion with at least 2 units of erythrocytes.^13^

High age was defined as 85 years of age or above. Multiple advanced comorbidities were defined as two or more of the following: Acute (stroke, acute myocardial infarction, sepsis, gastrointestinal bleeding, acute intestinal obstruction) or chronic (dementia, chronic obstructive pulmonary disease, chronic ischemic heart disease, previous stroke, heart failure, atrial fibrillation, metastatic cancer disease) conditions.

No run-off was defined as no patent arteries in the lower leg in magnetic resonance angiography, computed tomography angiography or duplex, or patent arteries in the lower leg, but signs of occlusion of foot arteries and pronounced decreased toe-brachial index. If there was an available echocardiographic assessment within a year prior to admission, severe cardiac failure was defined as ejection fraction <40%.^14^

### Ethics

This study has ethical clearance from the Swedish Ethical Review Authority (Dnr §2020-00764).

### Statistical Methods

The statistical analyses were carried out in Statistical Package for the Social Sciences (SPSS) Statistics version 26 (IBM, Armonk, NY, USA). Continuous variables were presented as median together with range or interquartile range (IQR), and inter-group differences were tested with the Mann-Whitney U test. Pearson’s chi-square test or Fisher’s exact test were used to analyze differences in frequencies between groups. The level of statistical significance was *P* < .05.

## Results

### Patient Characteristics Background in Relation to Initial Treatment

Patient and background data are shown in Table 1. Among 161 patients, 68 underwent endovascular and 20 open vascular revascularizations, whereas 73 (45.3%) were managed without revascularization. Conservative vascular therapy, primary amputation, and palliative care were conducted in 25 (15.5%), 26 (16.1%), and 22 (13.7%) patients, respectively. Patients treated with endovascular surgery (*P* < .001) were younger than those in the other groups, whereas patients undergoing primary amputation (*P* = .011) and palliative care (*P* = .021) were older than those in the other respective groups. Compared to men, median age for women was higher in the open vascular (*P* = .009) and conservatively (*P* = .013) treated groups. Endovascular therapy (*P* = .003) was more often performed in men, whereas palliative therapy (*P* = .004) was more often performed in women. Patients living in a nursing home vs not at admission underwent operative revascularization more seldom (*P* < .001) and more often underwent primary major amputation (*P* < .001). Compared to those living as cohabitants, living alone was more frequently occurring in those undergoing palliative therapy (*P* = .016). Atrial fibrillation was less often prevalent in those receiving endovascular therapy vs not (*P* = .002) and more often in those undergoing primary major amputation vs not (*P* = .002). History of claudication at admission was more often present in those undergoing endovascular therapy vs not (*P* < .001) and less often in those receiving conservative therapy vs not (*P* = .024). There were no differences in the rates of diabetes mellitus, ischemic heart disease, and cerebrovascular disease across the five groups. Aspirin use at admission was more common in patients undergoing endovascular therapy vs not (*P* = .010) and less common in patients receiving conservative therapy vs not (*P* = .039).

**Table 1.** Patients With Acute Lower Limb Ischemia in Relation to Initial Treatment.

Variable (n (%))	Endovascular	Open Vascular	Conservative Therapy	Primary Major Amputation	Palliative Care
	n = 68	n = 20	n = 25	n = 26	n = 22
Age (median, interquartile range)	71 (61-79)	72 (68-86)	77 (62-77)	78 (74-88)	86 (66-91)
Men	71 (60-79) (n = 44)	68 (52-74) (n = 9)	69 (58-76) (n = 12)	77 (66-88) (n = 12)	63 (58-88) (n = 5)
Women	71 (63-76) (n = 24)	80 (72-91) (n = 11)	85 (76-90) (n = 13)	82 (77-89) (n = 14)	89 (72-92) (n = 17)
Women	24 (35)	11 (55)	13 (52)	14 (54)	17 (77)
Living in a nursing home	1 (1.5)	0 (0)	2 (8.0)	8 (30.8)	3 (13.6)
Living alone	32 (47)	9 (45)	13 (57)	15 (58)	17 (77)
Arterial hypertension	50 (74)	18 (90)	22 (88)	19 (73)	16 (73)
Diabetes mellitus	23 (34)	4 (20)	5 (20)	8 (31)	5 (23)
Ischemic heart disease	16 (24)	5 (25)	4 (16)	8 (31)	8 (36)
Cerebrovascular disease	15 (22)	3 (15)	6 (24)	8 (31)	5 (23)
Atrial fibrillation	13 (19)	7 (35)	7 (28)	15 (58)	10 (45)
Intermittent claudication	40 (59)	7 (35)	5 (20)	7 (27)	6 (27)
Medical therapy					
Aspirin	34 (50)	8 (40)	5 (20)	7 (27)	8 (36)
Anticoagulation	16 (24)	2 (10)	6 (24)	6 (23)	3 (14)
<b>Laboratory status at admission</b>					
Renal insufficiency	21 (31)	10 (50)	16/22 (73)	11/25 (44)	15 (68)
Anemia	19 (28)	6 (30)	5/22 (23)	11 (42)	9 (41)
C-reactive protein (median, interquartile range) mg/L	6 (2 – 12) (n = 63)	8 (2 – 33)	7 (2 – 31) (n = 22)	78 (35 – 138)	45 (8 – 213) (n = 19)
Clinical status at admission					
Foot/leg ulcers	11 (16)	1 (5)	1 (4)	10 (38)	2 (9)
Rutherford ≥ IIb	17 (25)	14 (70)	8/24 (33)	19 (73)	14/18 (78)
Occlusion characteristics					
Supra-inguinal (as opposed to infra-inguinal occlusion)	25 (37)	11 (55)	2 (8)	4 (15)	8 (36)
Bypass graft occlusion	4 (6)	1 (5)	1 (4)	3 (12)	1 (5)
Endoprosthesis occlusion	23 (34)	4 (20)	0 (0)	1 (4)	0 (0)
Thrombus	25 (37)	6 (30)	6 (24)	7 (27)	7 (32)
Embolus	14 (21)	9 (45)	17 (68)	14 (54)	14 (64)
Popliteal artery aneurysm thromboembolic occlusion	2 (3)	0 (0)	1 (4)	1 (4)	0 (0)

### Patient Characteristics at Admission in Relation to Initial Treatment

Renal insufficiency was less prevalent in those undergoing endovascular therapy vs not (*P* < .001), and more prevalent in those undergoing conservative therapy vs not (*P* = .008), and palliative care vs not (*P* = .028). C-reactive protein levels were lower in patients treated with endovascular therapy vs not (*P* < .001), and higher in those undergoing primary amputation vs not (*P* < .001) and palliative care vs not (*P* = .001). Patients with foot ulcers were more often treated with primary major amputation vs not (*P* < .001). Patients with Rutherford class ≥ IIb ischemia underwent less often endovascular therapy vs not (*P* < .001), and more often open vascular surgery vs not (*P* = .022), primary major amputation vs not (*P* = .003), and palliative care vs not (*P* = .004). Supra-inguinal arterial occlusions were more often treated with open vascular surgery vs not (*P* = .013), whereas infra-inguinal arterial occlusions more often were treated conservatively vs not (*P* = .007). Endoprosthesis occlusions were more often treated endovascularly vs not (*P* < .001), and less often with primary major amputation vs not (*P* = .047), conservative therapy vs not (*P* = .013), or palliative care vs not (*P* = .021). Embolic occlusions were less often treated with endovascular therapy vs not (*P* < .001), but more often with conservative therapy vs not (*P* = .005), and palliative care vs not (*P* = .029) (Table 1).

### Outcomes in Relation to Initial Treatment

Rates of major amputation and mortality in the five groups at 30 days and one year are shown in Table 2. Compared to the other groups, patients treated conservatively did not have a lower major amputation rate at one year (*P* = .070). Patients undergoing primary major amputation vs not did not have a higher mortality rate at one year (*P* = .099).

**Table 2.** Outcomes in Patients With Acute Lower Limb Ischemia in Relation to Initial Treatment.

Variable (n (%))	Endovascular^a^	Open Vascular	Conservative Therapy	Primary Major Amputation	Palliative Care
	n = 68	n = 20	n = 25	n = 26	n = 22
Major amputation at 30 days	1 (1.5)	0	1 (4.0)	22 (84.6)	0 (0)
Major amputation at 1 year	5 (7.4)	2 (10.0)	2 (8.0)	26 (100)	0 (0)
Mortality at 30 days	2 (2.9)	3 (15.0)	1 (4.0)	4 (15.4)	19 (86.4)
Mortality at 1 year	6 (8.8)	6 (30.0)	6 (24.0)	12 (46.2)	22 (100)

^a^Three major amputations occurred after 1 year in the endovascular group.

### Reasons for Choosing Conservative Vascular Therapy

Twenty-five patients were treated conservatively. The reasons for choosing conservative treatment were often multiple (Table 3). The median age was 88 years (range 85 – 97) among those judged to be of high age. Four of these patients had another acute disease than ALI: synchronous arm embolism (n = 1), synchronous cerebral embolism and acute myocardial infarction (n = 1), septicemia (n = 1), and mechanical colonic ileus due to obstructive cancer (n = 1). There were two additional patients with cancer, one with malignant melanoma (awaiting operation) and one with pulmonary cancer (newly diagnosed). All three patients with cancer disease were newly diagnosed without evidence of metastatic disease at diagnosis. Two of these patients died due to cancer-related disease; one due to acute colonic obstruction (due to cardiac failure and catabolism after successful endoluminal metallic stent implantation in the sigmoideum), and one due to metastatic pulmonary cancer at 3 years of follow-up including oncological treatment.

**Table 3.** Contributory Reasons for Choosing Conservative Therapy, Primary Major Amputation or Palliative Care in Patients With Acute Lower Limb Ischemia.

Variable	Frequency (n)		
	Conservative Therapy	Primary Major Amputation	Palliative Care
	n = 25	n = 26	n = 22
Patient-related factors			
High age (≥85 years)	7	10	12
Multiple advanced comorbidities	7	15	11
Prevalent cancer disease	3	2	3
Dementia	2	4	4
Severe cardiac failure (ejection fraction <40%)	3	4	7
Illicit drug abuser	2	1	
Previous contralateral femur amputation	1	1	
Wheel-chair dependent	1	2	1
Refuses any operation	-	-	2
Refuses vascular surgery		2	
Refuses amputation		-	1
Non-communicative		4	3
Disease-related factors			
Pronounced motor deficit		12	4
Advanced gangrene of the foot		5	
Foot artery embolism	3		
Synchronous embolism	2		
Concomitant ipsilateral acute deep venous thrombosis	2	2	
No run-off	1	1	2
Abdominal aortic aneurysm	1		
Aortic occlusion			3
Occluded bypass with previous multiple reinterventions	1	2	
Occluded stentgraft in the poplitea for popliteal aneurysm and no vein available		1	
Septicemia/erysipelas due to necrotic toes	1	1	1
Septic shock with focus foot wound		1	
Foot artery thrombosis	1		
Acute myocardial infarction	1		
Pathological-anatomical factors			
Advanced revascularization procedure (femoro-crural bypass) would have been required	2		
Treatment-related factors			
Initial clinical response to anticoagulation therapy	10		
Exploration of calf artery only – unsuitable for bypass		1	
Orthopedic consultation		26	7
Bilateral major amputation necessary			1
Documented treatment restrictions in journal prior to admission		1	

The sum exceeds total number of patients in each group since there often were multiple reasons per patient.

Two patients chosen for conservative therapy would have required an advanced revascularization procedure (femoro-crural bypass). One of these patients had an ejection fraction <25%.

### Conservative Vascular Therapy

Initial treatment at discharge consisted of enoxaparin (n = 13), dalteparin (n = 7), apixaban (n = 2), dual antiplatelet treatment with aspirin and clopidogrel (n = 1), or aspirin alone (n = 1). One patient with an occluded femoro-popliteal bypass did not receive any anticoagulation or analgesics at all. Full-dose enoxaparin for up to six weeks was given to five patients, and an indeterminate half-dose regimen of enoxaparin was given to eight patients. After enoxaparin treatment, anti-platelet therapy with aspirin or clopidogrel was given to seven patients and peroral anticoagulation (apixaban, rivoraxoban) to two. Dalteparin was given in full dose to seven patients, followed by peroral full-dose anticoagulation (rivaroxaban [n = 4] or vitamin K antagonist) in five, and aspirin in two. Three patients treated with full dose dalteparin had concomitant deep venous thrombosis. One patient with ALI due to acute thrombotic occlusion of a popliteal artery aneurysm underwent a femoro-popliteal bypass after five weeks of full-dose treatment with apixaban.

Analgesics was given to twelve patients at discharge. Ten patients were prescribed paracetamol in doses ranging from one tablet (.5 g) if necessary to a full-dose regimen (1 g four times daily). Opiates were prescribed to six patients, in doses ranging from oxikodon 5 mg if necessary to a combination of oxikodon and naloxon 5 mg/2.5 mg mg twice daily.

Four patients had embolic foot embolization with median ankle brachial index of .9 (range .7 – 1.2). Absence of acute occlusions above the ankle was confirmed by computed tomography angiography or magnetic resonance angiography. One patient with foot artery embolism was treated conservatively for twelve weeks before undergoing stenting of an underlying common iliac artery stenosis. There were no amputations or mortality at one year in this subgroup of conservatively treated patients.

One patient had a major bleeding complication. The patient had blood in his stools one day prior to admission and deteriorated with bloody vomiting and more rectal bleeding after initiation of enoxaparin 60 mg subcutaneous once daily. He received seven units of erythrocytes, two units of plasma, one unit of thrombocytes, and treatment with esomeprazole. Upper endoscopy diagnosed grade C (severe) esophagitis. Administration of enoxaparin and clopidogrel was discontinued. After four days, a reduced dose of enoxaparin 40 mg subcutaneous once daily was reinstalled for six days. At discharge, clopidogrel was reinstalled and enoxaparin discontinued permanently.

### Reasons for Choosing Primary Major Amputation and Palliative Care

The three most common reasons for choosing primary major amputation were multiple advanced comorbidities, high age and pronounced motor deficit in the leg, and the three most common reasons for choosing palliative care was high age, multiple advanced comorbidities, and severe cardiac failure (Table 3). Four patients underwent major amputation beyond 30 days: after 35, 39, and 41 days, and after four months. This last patient had an asymptomatic popliteal artery aneurysm and was treated by endovascular stentgraft repair due to absence of suitable vein material for bypass. The stentgraft occluded with onset of ALI and there was no other treatment option than primary major amputation. There was a transition from ALI to chronic limb-threatening ischemia with progressive toe necrosis within a month and then gradually worsened pain status ending up with acceptance of major amputation. Two patients in the primary major amputation group had metastatic pulmonary cancer.

There were seven patients that crossed over from major amputation to palliative care. Among those 22 patients receiving final palliative care, seven (32%) had an orthopedic consultation. The requests for major amputation were turned down in five patients and two scheduled major amputations were cancelled. There were three patients in the palliative care group with metastatic cancer disease (pulmonary, breast, and colonic cancer).

### In-Hospital Management of All 38 Patients Undergoing Major Amputation

The in-hospital management of all 38 patients undergoing major amputation is shown in Table 4. After 13 re-amputations, the final levels of amputation were at the upper and lower leg in 27 (71%) and 11 (29%) patients, respectively. Twenty (53%) patients had wound infection at the site of amputation. Seventeen wound cultures were taken, and 16 were positive. Nine patients had growth of *Staphylococcus aureus*, and 13 cultures showed growth of any intestinal bacterial species. Twelve patients had more than one cultured bacterial species. Morbidity related to the amputation stump and short-term mortality are shown in Table 4.

**Table 4.** In-Hospital Management of all 38 Patients Undergoing Major Amputation.

Variable	Frequency (n (%))
	n = 38
Level of major amputation (including any re-amputation)	
Upper leg	27 (71)
Lower leg	11 (29)
Re-amputation	13 (34)
Problem related to amputation stump	22 (58)
Postoperative wound infection of the amputation stump	20 (53)
Revision of amputation stump	15 (39)
In-hospital stay (median, inter-quartile range) related to the amputation procedure; days	34 (20 – 63)
30-day mortality	4 (11)
In-hospital mortality	7 (18)

### Evaluation for Limb Prosthesis

Among 31 patients discharged from the hospital, 21 were considered for potential use of prosthesis, of whom 14 were deemed suitable for prosthesis fitting and received a limb prosthesis. The main reasons for not qualifying for a limb prosthesis were advanced dementia, previous walking inability, contralateral ischemic foot wound, and short amputation stump (Table 5).

**Table 5.** Evaluation for Limb Prosthesis.

Variable	Frequency (n)
Patients discharged after major amputation	31
Evaluation for use of limb prosthesis at the outpatient clinic	21
Admitted to facility for walking with limb prosthesis	14
Limb prosthesis received	14
Lower leg amputated	7/11
Upper leg amputated	7/27
Reasons to not qualify for limb prosthesis (n = 17)^a^	
Fatal pneumonia post-discharge	1
Advanced dementia	5
Not able to walk	4
Chronic wound healing disturbance/infection in amputation stump	2
Short amputation stump	3
Contralateral ischemic limb/foot wound	4
Too weak in the contralateral limb	1
Too poor balance	1
Alcoholism	1
Metastatic cancer disease	1
Unmotivated	1

^a^Some patients had multiple reasons why the sum of reasons exceeds 17.

## Discussion

The meticulous search methodology in this population-based study enabled us to show that a substantial proportion of patients with ALI, 45%, did not undergo operative revascularization. Sweden is a high-income country, and the vascular center in Malmö is located centrally in the city. This busy high-volume unit with services around the clock has for decades primarily been oriented towards minimal invasive endovascular surgery,^15,16^ and local thrombolytic therapy has been the mainstay of therapy for ALI in the new millennium.^15^ This endovascular profile as opposed to open vascular surgery means that more elderly fragile patients with high comorbidity burden might be eligible for operative treatment of ALI. As we previously did not find any out of hospital deaths from ALI in this population,^7^ patients with ALI are contemporarily admitted to hospital in time for an assessment. Despite all these factors facilitating operative treatment for ALI, a large proportion of patients in our study were not treated by operative revascularization. We believe that the meticulous search in hospital charts of all patients with a possible diagnosis of ALI retrieved from the different patient information systems contributed to the finding of a high proportion of non-operatively treated patients.

High age, advanced stage of ALI, and increased C-reactive protein levels at admission, indicating a more severe ALI, were identified as factors associated with primary major amputation and palliative care, and living in a nursing home, atrial fibrillation, and foot ulcers were other factors more often present in patients receiving primary major amputation. Interestingly, the proportion of previous diagnoses such as diabetes mellitus, ischemic heart disease, or cerebrovascular disease did not differ between groups, which means that comorbidity burden appears to have a comparably low influence on the initial treatment decision. Of major concern, though, was the report on the association between inadequate physical examination by the first doctor at the emergency department, resulting in delays in diagnosis and treatment, and lower amputation-free survival in this population.^17^

Evaluation of primary conservative therapy in ALI, mainly with anticoagulation alone, has indeed been scarcely reported in the literature, particularly in adult patients, and only small case series are available.^18^ The reasons for choosing primarily anticoagulation therapy in our study were often multiple as shown in Table 3. Detailed interpretation of medical records showed that this decision could often be attributed to patient- and disease-related factors and initial clinical response to anticoagulation therapy, whereas pathological-anatomical factors and complexity of the required revascularization were found to be less important. The present study showed that 16% of patients were treated by primary conservative vascular therapy alone. It should be noted that Rutherford class IIb ischemia was present in one-third of these patients at admission, a slightly higher proportion than in patients treated with endovascular methods. This conservatively treated subgroup of patients had higher rates of renal insufficiency, infra-inguinal arterial occlusions, and embolic occlusions, and there was also a non-significantly lower rate of major amputations at one year. There were four patients with foot embolization who all had favorable outcomes. The C-reactive protein levels at admission in this subgroup of conservatively treated patients were low, which appeared to reflect a more favorable prognosis.^19^

**Figure 1.**
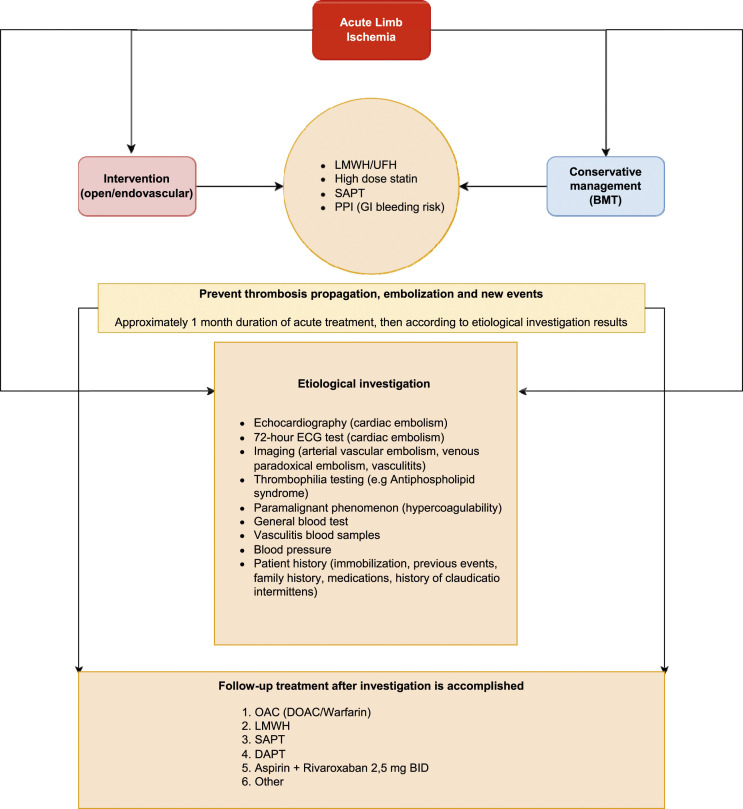
A proposed algorithm for conservative management and diagnostic work-up in patients with ALI. LMWH; low-molecular weight heparin, UFH; unfractionated heparin, SAPT; single-antiplatelet therapy, PPI; proton pump inhibitors, BMT; best medical treatment, ECG; electrocardiogram, OAC; oral anticoagulation, DOAC; direct oral anticoagulants, DAPT; dual antiplatelet therapy, BID; “bis in die” (latin meaning “twice daily”).

Initial medical treatment with full-dose unfractionated heparin, initiated by a bolus dose followed by infusion, is recommended for patients with ALI awaiting operative revascularization in the European Society of Vascular Surgery (ESVS) Guidelines^10^ to reduce further embolism or clot propagation, whereas there is no guideline support for continued systemic therapeutic heparinization after thrombolysis.^10^ Meta-analysis^20^ from the randomized COMPASS^21^ and VOYAGER^22^ trials have shown a reduction of ALI in patients receiving low-dose rivaroxaban, 2.5 mg twice daily in combination with low-dose aspirin, both in stable peripheral artery disease and immediately after revascularization. These treatment strategies, initial full-dose of unfractionated or low-molecular weight heparin after diagnosis and combination therapy of low-dose direct oral anticoagulants and single antiplatelet regime may perhaps be beneficial for those primarily conservatively treated for ALI as well. The anticoagulation therapy was heterogenous in the present study, ranging from half to full-dose low-molecular weight heparin for six weeks followed by peroral indeterminate anticoagulation. Only half of conservatively managed patients were treated with low dose of analgesics at discharge. The positive outcomes of these conservatively treated patients need to be better evaluated in a multicenter collaboration with a predefined protocol, in which the dose of anticoagulation and duration of treatment need to be fixed, whereas analgesic therapy should be individualized. An algorithm for conservative medical management and diagnostic work-up in patients with ALI is proposed in Figure 1.

Among patients undergoing major amputation after hospital discharge, 45% were fitted with a prosthesis. As expected,^23^ there was a higher percentage of prosthesis use in those with a lower than upper leg amputation. The postoperative wound infection rate of the amputation stump was high; 53%. This wound infection rate is slightly higher than the 40% reported by Berridge et al.^24^ The high re-amputation rate and revision of the amputation stump of 34% and 39%, respectively, indicate, that there was presence of residual ischemia and severe disturbances of wound healing in the present study on patients exclusively suffering from ALI. It cannot be excluded that a few of these amputation stumps would have healed better if prior revascularization had been attempted. There is certainly room for improvement in this area, and a recently published randomized trial using incisional negative pressure wound therapy has shown a reduction of incidence of surgical site infection and improved stump healing.^25^ Hopefully, there will be consistent results in similar future randomized trials in patients undergoing major amputation due to ALI.

The small sample size with no adjustment for confounding and selection bias constitutes important limitations of the study. Larger prospective studies with predefined protocols of anticoagulation treatment are warranted to better define the role of initial conservative therapy in patients with ALI receiving anticoagulation alone. A strength of the study, on the other hand, is the population-based sample covering all retrieved patients with ALI within a defined population enabling analysis of subgroups not undergoing operative revascularization.

These results should be interpreted with caution. An optimal candidate for conservative therapy has no pronounced motor deficit, no advanced gangrene of the foot, and initial clinical response to anticoagulation therapy. The preferred patient should also have foot thromboembolism alone and low CRP at admission. Conservative therapy might be considered as first line in patients with synchronous embolism and when an advanced revascularization procedure is required in an elderly fragile patient. Patients considered for conservative vascular therapy after an ALI event should be followed up initially by a vascular physician or vascular surgeon. In a patient with foot artery thromboembolism, investigation of toe-brachial index at discharge and first follow-up visit is indicated.

In conclusion, ALI patients selected for initial conservative therapy with anticoagulation may have a good outcome, even when admitted with Rutherford class IIb ischemia. Low C-reactive protein levels at admission seem to be a favorable prognostic marker in patients undergoing conservative therapy.
